# How data visualization elevates nursing performance insight

**DOI:** 10.1097/nmg.0000000000000355

**Published:** 2026-02-27

**Authors:** Angela Pascale

**Affiliations:** **Angela Pascale** is a Research Analyst at Press Ganey Associates LLC in South Bend, IN.

**Figure:**
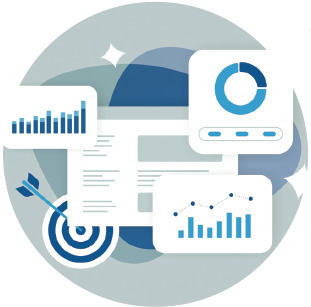
No caption available.

*This article is the first in a four-part series on effectively visualizing, interpreting, and communicating data to clearly demonstrate nursing's impact on quality, safety, and financial outcomes. This series is intended to provide nurse leaders with the skills to transform raw data into compelling stories that inform strategic decisions and elevate nursing's contribution across the organization*.

**NURSE MANAGERS (NMS)** are accountable for a wide range of performance outcomes that directly influence patient safety, staffing stability, and resource allocation. Yet, the data behind these outcomes often live across multiple platforms, making it hard to see the full picture. Data visualization addresses that challenge by transforming scattered metrics into clear, actionable insights—helping NMs quickly recognize patterns, spot priorities, and connect performance trends to operational decisions. Instead of sifting through raw numbers, visualization brings meaning forward, enabling leaders to act with confidence and precision. This column establishes why visualization matters and how it elevates NMs' practice.

## THE VALUE OF VISUALIZING NURSING PERFORMANCE DATA

NMs are accountable for a wide range of performance information such as the National Database of Nursing Quality Indicators® (NDNQI®), staffing measures, and other unit-level quality metrics. These metrics provide essential insights into clinical outcomes, staffing patterns, and workforce performance. Visualizations build on the information NMs already receive by illuminating patterns, changes, and priorities. When a visualization highlights a clear pattern—whether in trends, variation, or benchmarks—this awareness helps NMs move from simply viewing raw data to determining what requires attention visually. Visualization is the bridge from reporting to insight.

Importantly, visualization adds essential context to performance data by revealing patterns that are difficult to see in raw numbers. Well-designed visuals help NMs see how different categories of performance, including staffing, quality and workflow metrics, relate and align across shifts or time periods. Visuals show whether values appear stable or shifting, and whether a recent change in staffing or workflow or the introduction of a new intervention coincides with a noticeable change in performance measures. These visual cues—such as shifts in direction, changes in magnitude, or contrasts between groups—help make sense of patterns, relationships, and variations in the data that are most important for understanding unit needs and setting priorities.

This clearer picture becomes especially valuable when developing a nursing business case. Whether proposing a virtual nursing model, strengthening fall-prevention strategies, or requesting support roles, NMs often need to demonstrate how unit-level performance aligns with the resources they're seeking. Visualization helps make that link visible by showing how changes in practice, staffing, or workflow connect to shifts in outcomes. A focused display—such as a before-and-after view or side-by-side comparison—can help articulate the clinical rationale, describe anticipated improvements, and convey potential return on investment. Visuals also support cross-disciplinary conversations by presenting information in a format that clinical, financial, and executive partners can easily use and engage with.

## HOW VISUALIZATION DEEPENS UNDERSTANDING

Visualization brings forward the story within performance data. Reports provide essential values on which NMs rely, and visual displays build on that foundation by showing how those values move, cluster, or change over time. When displayed visually, patterns that support clinical and operational decisions become easier to recognize at a glance.

Visualization of data helps:

- **detect trends over time and spot emerging priorities,** such as a steady month-to-month rise in fall rates that signals a need for early intervention, using a run chart;
- **identify variation across units, teams, or shifts**, such as seeing that night shifts have consistently higher falls than day shifts, using a grouped bar chart;
- **understand distribution and spread within a measure**, such as identifying isolated shifts where RN skill mix drops sharply compared with the usual pattern, using a stacked bar chart;
- **reveal relationships between measures**, such as visualizing how pressure injuries increase during weeks when RN skill mix is lower, using a combined line-and-bar graph; and
- **connecting performance to staffing,** workflow, or recent interventions, such as observing that falls declined after proposal rounding was implemented, using an intervention line on a run chart.

**FIGURE 1:**
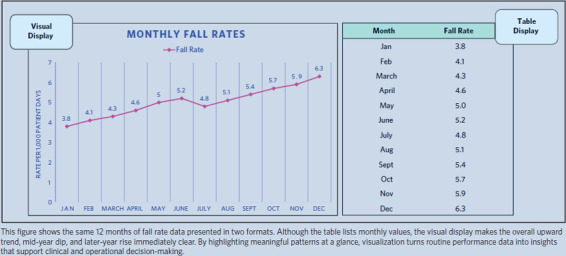
Visual display reveals trends that are hard to see in tables

Visualization provides a clearer view of performance by making key patterns, differences, and relationships easier to recognize at a glance. For example, Figure 1 shows how the same 12 months of fall-rate data look in a visual display versus a table, illustrating how trends become easier to recognize when shown graphically. Recognizing how the same data can look different depending on the display also underscores why visualization choices matter—certain formats can make patterns easier to see, whereas other formats may obscure them.

## COMMON PITFALLS IN NURSING DATA

Visual displays are meant to clarify information, but when key design principles are overlooked, important patterns can become harder to recognize. The following common pitfalls illustrate how display choices can affect clarity and interpretation.

1. ***Information overload***. Displaying too many metrics or dense month-by-month values in a single visual can obscure meaningful patterns.
2. ***Inconsistent scaling or indistinguishable color use***. Using different Y-axis ranges across metrics, or colors that are too similar, makes it difficult to compare values or distinguish one measure from another. Aligned scales and clearly differentiated colors support visual accuracy and pattern recognition.
3. ***Overuse of bar charts when trends matter***. Relying on bar charts to display monthly or quarterly performance can hide how trends are evolving. When data are collected multiple times per year, line-based visuals make it easier to interpret changes over time and identify patterns.
4. ***Ambiguous or incomplete labeling***. Omitting clear axis labels, time frames, or metric definitions makes it harder to understand what's being measured or how to interpret changes over time.
5. ***Missing benchmarks or targets***. Presenting visuals without comparison points—such as unit goals, national benchmarks, or organizational targets—limits the ability to determine whether performance is improving, is stable, or requires action.

**FIGURE 2:**
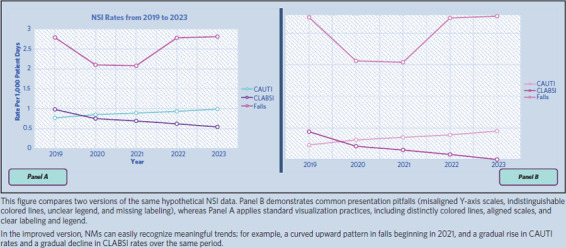
Impact of presentation choices on visualizing nursing performance data

These challenges arise not from the data but from how the data are presented. Figure 2 illustrates how design choices—such as scaling, color use, and labeling—can either conceal important trends or make them easier to recognize. By addressing these issues, NMs can more clearly interpret their unit's performance and communicate insights with greater precision.

## THE NUMBERS-TO-NARRATIVE MODEL

Nurse managers are accountable for outcomes that influence safety, staffing, and resource allocation. The Numbers-to-Narrative Model helps transform routine metrics into actionable insights by pairing visualization with interpretation—bridging the gap between raw data and operational decisions.

**FIGURE 3:**
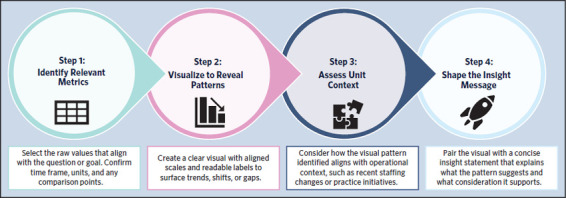
The Numbers-to-Narrative Model: A four-step process for turning nursing metrics into actionable insight

Turning data into insight requires more than simply displaying numbers; it requires understanding what the visual suggests and how it connects to operational context. Visual displays clarify what's happening in the data, but they don't independently explain why this knowledge matters or what action this insight should inform. The Numbers-to-Narrative Model (described in Figure 3) provides a practical framework that helps NMs translate routine performance metrics into clearer, evidence-informed insights, strengthening the ability to communicate key performance needs across leadership and support the development of a compelling business case for nursing initiatives.

### Step 1: Gather raw metrics

NMs regularly receive values, such as monthly rates, percentages, counts, benchmarks, and targets related to care quality, staffing, and workflow. On their own, these metrics show performance but don't clearly reveal patterns or operational meaning. This step focuses on organizing which raw values and comparison points are available, so the information can later be used to build a visual and determine how emerging patterns relate to operational needs, resource considerations, or the development of a business case.

### Step 2: Visualization and pattern recognition

This step involves transforming the selected raw values from Step 1 into a visual format that makes the underlying story easier to see. By displaying the data graphically, patterns such as trends, shifts, variation, gaps from targets, differences between units, or the potential influence of a staffing change or intervention become more apparent.

### Step 3: Assess operational implications

This step examines how the visual pattern connects to what's happening on the unit. Operational information—such as recent staffing adjustments, workload changes, workflow constraints, practice initiatives or shifts in patient needs—is reviewed alongside the pattern to understand its possible relevance. By considering how the pattern aligns with these real-world conditions, it becomes easier to identify potential implications for staffing ratios, workflow efficiency, care processes, effectiveness of an intervention, or resource requirements. This clarification helps determine whether the observed pattern and visual contributes meaningful support for developing a business case or making a focused request for resources.

### Step 4: Shape the narrative for action

This step translates the interpreted pattern into a clear, concise, evidence-informed message that pairs with the visual display. The goal is to clearly articulate what the pattern suggests, why it matters for the unit, and what action or consideration it supports. When presented alongside the visual, this short narrative enhances clarity; strengthens the ability to communicate key points; and helps frame the insight for business-case development, resource needs, or performance improvement discussions. Applying this model strengthens the business case for nursing initiatives by making performance patterns clearer to interpret and communicate, enabling NMs to more easily connect clinical and workforce patterns with organizational priorities and resource decisions.

## REAL-WORLD EXAMPLE: TRANSLATING NDNQI DATA INTO INSIGHT USING VISUALIZATIONS

To show the Numbers-to-Narrative model process in practice, this section applies the four-step process to a set of commonly used NDNQI nursing performance measures. In many organizations, NDNQI indicators are among the most utilized sources of unit-level performance information. These metrics capture essential aspects of nursing care—such as falls, pressure injuries, or staffing measures—and offer a structured way to monitor performance over time. The downloadable PDF supplement illustrates how turning the same data into a visual format can surface patterns and relationships more clearly (see *Supplementary Content, available online at* http://links.lww.com/NMT/A10). Using NDNQI fall-rate and RN-hours-per-patient-day data, this supplement provides a practical example of how the Numbers-to-Narrative process can be applied to routine unit-level metrics. This example reinforces how a structured, visual approach can help NMs move from routine reporting to clearer, more actionable insights that support informed, unit-level decision-making.

## CONFIDENCE AND QUALITY CARE

Data visualization gives NMs a clearer, more meaningful view of performance by transforming routine metrics into recognizable patterns and actionable insights. Using the Numbers-to-Narrative Model, these visuals help leaders connect trends to operational realities, strengthening communication and decision-making. By adopting a structured, visual approach, NMs can more confidently interpret unit needs, advocate for resources, and drive improvements that support safe, high-quality care.

